# Bioinformatics analysis of the prognostic and clinical value of senescence-related gene signature in papillary thyroid cancer

**DOI:** 10.1097/MD.0000000000033934

**Published:** 2023-06-02

**Authors:** Tingting Wen, Shuang Guo

**Affiliations:** a Department of Vascular and Thyroid Surgery, The First Hospital of China Medical University, Shenyang, China; b Department of Internal Medicine-Oncology, The First Hospital of China Medical University, Shenyang, China.

**Keywords:** bioinformatics analysis, clinical value, papillary thyroid cancer, prognostic, senescence

## Abstract

Cellular senescence can both inhibit and promote the occurrence of tumors, so how to apply cellular senescence therapy is of great importance. However, it is worth to be analyzed from multiple perspectives by researchers, especially for tumors with a high incidence like papillary thyroid cancer (PTC). We obtained senescence-related differentially expressed genes (SRGs) from The Cancer Genome Atlas (TCGA) and gene expression omnibus database. Enrichment analysis of SRGs was performed via gene ontology and Kyoto Encyclopedia of Genes and Genomes. Prognostic model was constructed by univariate and multivariate Cox regression analysis. Evaluation of clinical value was analyzed via Receiver operating characteristic curve, Kaplan–Meier curve and Cox regression. Immune infiltrates were investigated through ESTIMATE and single-sample gene set enrichment analysis. Immunohistochemical images were obtained from The Human Protein Atlas. Twenty-seven SRGs from TCGA cohort and gene expression omnibus datasets were found. These genes are mainly concentrated in senescence-related terms and pathways, including “DNA damage response, signal transduction by p53 class mediator,” “signal transduction in response to DNA damage,” “p53 signaling pathway” and “Endocrine resistance.” Based on SRGs, prognostic model was constructed by E2F transcription factor 1, snail family transcriptional repressor 1 and phospholipase A2 receptor 1. PTC patients were divided into a low-risk group and a high-risk group according to the median value (cutoff point = 0.969) of risk score in TCGA cohort. The diagnostic efficiency of this model is good (area under curve = 0.803, 0.809, and 0.877 at 1, 2, and 3 years in TCGA; area under curve = 0.964, 0.813 in GPL570 and GPL96), particularly advanced grade, state and tumor mutation burden, such as Stage III − IV, T3 − 4, H-tumor mutation burden. Furthermore, High-risk group was significantly associated with poor prognosis and more immune infiltration. Our prognostic model has a good diagnostic and prognostic efficacy, and there is a certain clinical application value. In addition, we provide the first new insight into the genesis, diagnosis, prognosis and treatment of PTC based on senescence-related genes.

**Key points**

• Analysis of the occurrence and development of thyroid cancer (PTC) from the perspective of senescence.
• Senescence-related abnormal genes may represent unique diagnostic and therapeutic targets as indicators of PTC.
• We constructed a PTC risk model based on senescence-related genes that are closely associated with immune cell infiltration and may be potential immunotherapeutic targets.

## 1. Introduction

Thyroid cancer (THCA) is the most common endocrine malignant tumor worldwide,^[1]^ accounting for 3.4% of all cancers diagnosed annually.^[2]^ The incidence of THCA has increased in the past 3 years, driven mainly by new cases of papillary thyroid cancer (PTC).^[2]^ PTC is the most common histological subtype of THCA, accounting for 90% of new cases, and generally has a good prognosis,^[3,4]^ but the incidence of cervical lymph node metastasis is high, which can easily lead to local recurrence and bring poor prognosis to patients.^[5]^ Therefore, it has become an important research topic to effectively identify specific patients and provide personalized treatment.

Ionizing radiation is the first risk factor for THCA, especially in childhood and adolescence.^[2]^ ionizing radiation can induce normal cells to enter the senescence state, resulting in normal tissue fibrosis and organ dysfunction, and even the development of cancer.^[6,7]^ Senescent cells have the ability to promote and mitigate the dual effects of cancer.^[8,9]^ On the 1 hand, cellular senescence essentially inhibits tumorigenesis of pretumor cells; on the other hand, the senescence-associated secretory phenotype generated by senescent cells can actually promote tumor growth, recurrence, and metastasis externally.^[10–12]^ Therefore, actively exploring the key role of senescence-related genes in PTC will provide new ideas and targets for the diagnosis, treatment and prevention of PTC patients.

In our study, the cancer genome atlas (TCGA) cohort was combined with 2 gene expression omnibus (GEO) datasets (GPL570 and GPL96) to explore potential differentially expressed genes (DEGs), and then combined with senescence-related genes to construct a prognostic risk model. On the basis of this model, the significance and value of its clinical application were analyzed, including diagnosis, prognosis and even prognosis of different subtypes of clinical parameters. In addition, the crosstalk between senescence and immune cell infiltration was preliminarily analyzed.

## 2. Materials and methods

### 2.1. Data extraction

The THCA dataset, containing normal thyroid samples (N = 59) and PTC samples (T = 511), was obtained from TCGA (https://tcga-data.nci.nih.gov/tcga/). The normalized RNA-seq data (FPKM), single nucleotide variation data (VarScan) and clinical data were downloaded from the TCGA data portal (see Table S1, Supplemental Digital Content, http://links.lww.com/MD/J79, which lists baseline data of papillary thyroid cancer patients from the The Cancer Genome Atla database).

Meanwhiles, the other 8 PTC microarray datasets were acquired from the GEO database (http://www.ncbi.nlm.nih.gov/geo): GSE3467 (N = 9, T = 9), GSE3678 (N = 7, T = 7), GSE33630 (N = 45, T = 49), GSE53157 (N = 3, T = 15) and GSE60542 (N = 30, T = 33) derived from GPL570 platform, GSE5364 (N = 16, T = 35), GSE27155 (N = 4, T = 51) and GSE58545 (N = 18, T = 27) came from GPL96 platform. According to different platforms, we combined the microarray datasets for subsequent research, called GPL570 and GPL96.

Furthermore, 279 senescence-related genes were downloaded from The Cellular senescence Gene Database (https://genomics.senescence.info/cells/) (see Table S2, Supplemental Digital Content, http://links.lww.com/MD/J80, which lists 279 senescence-related genes downloaded from The Cellular senescence Gene).^[13]^

The study of our project does not involve the detection of Human specimens. The immunohistochemical staining data comes from the online database of the human protein atlas, so it does not need the approval of the Ethics Committee.

### 2.2. Differential expression analysis

The DEGs were identified by performing the limma R package in R studio (version 4.1.1, https://posit.co/download/rstudio-desktop/) with the statistical threshold of |logFC| > 1 and false discovery rate < 0.05.^[14]^ We find the intersection of DEGs from GPL570, GPL96 and TCGA with senescence-related genes to obtain senescence-related DEGs (SRGs).

### 2.3. Gene functional enrichment analysis

Next, Gene Ontology (GO) and Kyoto encyclopedia of genes and genomes (KEGG) pathway enrichment analysis were performed for these SRGs with *P* < .05 and false discovery rate < 1 by using the “cluster Profiler” R package.^[15,16]^

### 2.4. Construction of a senescence-related risk model

We further analyzed SRGs to construct a senescence-related prognostic model. Univariate Cox regression analysis was performed to identify possible prognosis-related genes. Multivariate Cox regression analysis was applied to build prognostic model for PTC patients. Then, the risk score of the model was calculated using the following formula: score = e^sum (each gene’s expression × corresponding coefficient)^. Accordingly, PTC patients were classified into low-risk and high-risk groups by using the median value of risk score as the cutoff point.

### 2.5. Diagnostic and prognostic value analysis

Receiver operating characteristic (ROC) curves were drawn and the area under the ROC curve (AUC) was calculated by using the “survival ROC” R package according to the cutoff point of risk score and survival status of each patient. Kaplan–Meier (K-M) curves were plotted using “survminer” R package to compare overall survival (OS) between low-risk and high-risk groups.^[17]^

### 2.6. Clinical parameters analysis

Univariate and multivariate Cox regression analysis was performed to identify the prognostic value of clinical parameters and risk scores for PTC patients. Besides, stratified analysis of clinical parameters further explored differences in OS between low-risk and high-risk groups.

### 2.7. Tumor immune infiltration analysis

We used ESTIMATE to evaluate the immune cell infiltration level (immune score), matrix content (stromal score) and comprehensive score (ESTIMATE score) in PTC sample, and we compared the tumor microenvironment for PTC patients in low-risk and high-risk groups.^[18]^ The infiltrating score of 16 immune cells and the activity of 13 immune-related pathways were performed by the “single-sample gene set enrichment analysis” function in “gsva” R package.^[19]^

### 2.8. Immunohistochemistry analysis

Finally, we compared the differences in expression levels of E2F transcription factor 1 (E2F1), snail family transcriptional repressor 1 (SNAI1) and phospholipase A2 receptor 1 (PLA2R1) between normal and thyroid cancer tissues by analyzing immunohistochemistry images from the human protein atlas (https://www.proteinatlas.org).^[20]^

### 2.9. Statistical analysis

Mann-Whitney test was conducted to compare data between different groups. Pearson correlation analysis was used to explore relationships among the variables. Chi-square test was performed to analyze the differences of clinical parameters between the low-risk and high-risk groups. Statistical analysis and data visualization were performed by using R studio software (V.4.1.1) and GraphPad Prism (V.8.0). *P* < .05 was considered statistically significant.

## 3. Results

### 3.1. Identification of SRGs

**Figure 1.**
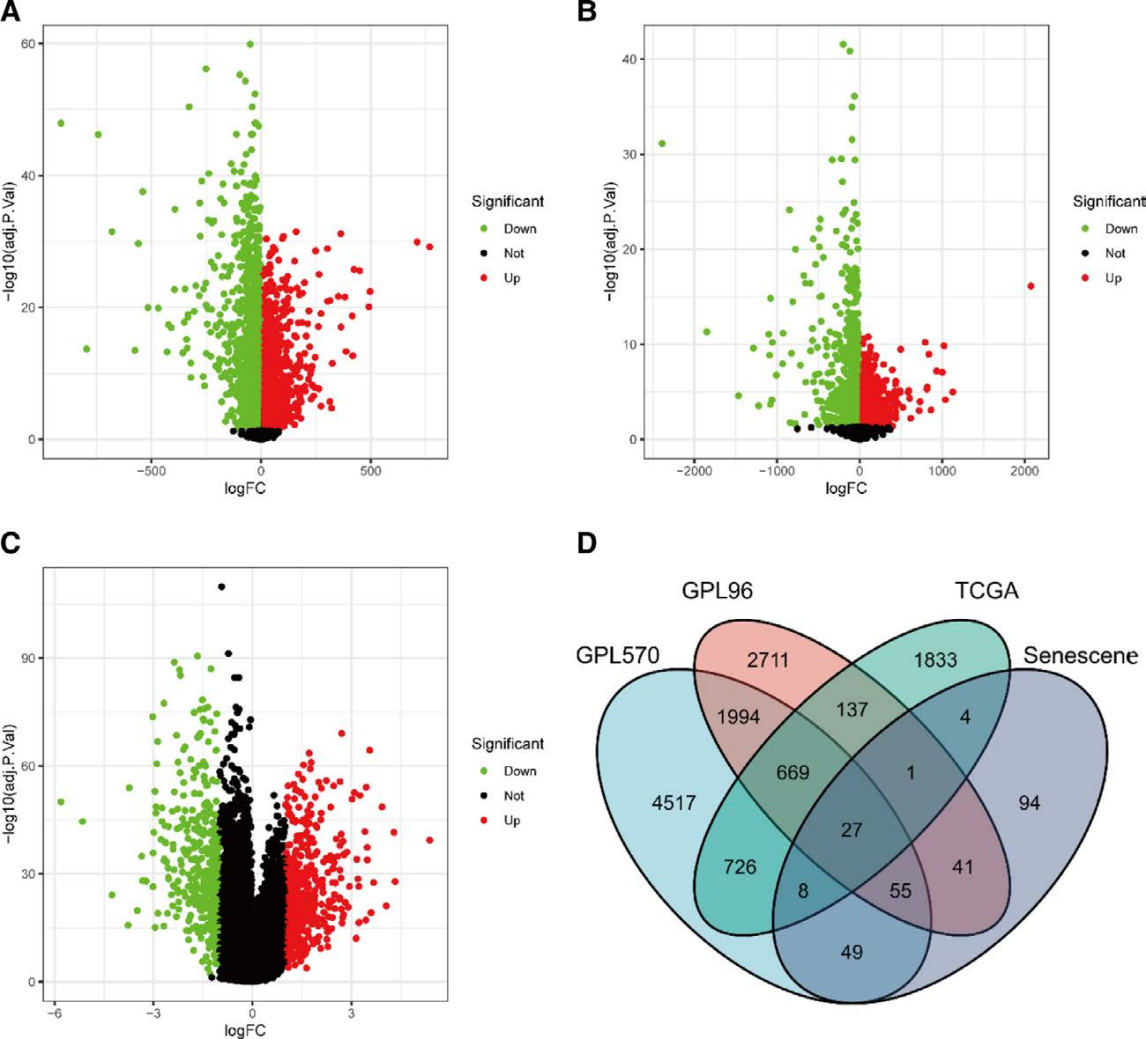
Identification of senescence-related differentially expressed genes (SRGs).

First, DEGs were screened out from GPL570, GPL96 and TCGA respectively (see Table S3, Supplemental Digital Content, http://links.lww.com/MD/J81, which lists 27 overlap genes of GPL570, GPL96, The Cancer Genome Atlas and senescence-related genes). DEGs were shown in the form of Volcano Plot (Fig. 1A–C). Then we observed 27 overlap genes of these DEGs with 279 senescence-related genes in Venn Diagram (Fig. 1D).

### 3.2. Gene functional enrichment analysis of SRGs

**Figure 2.**
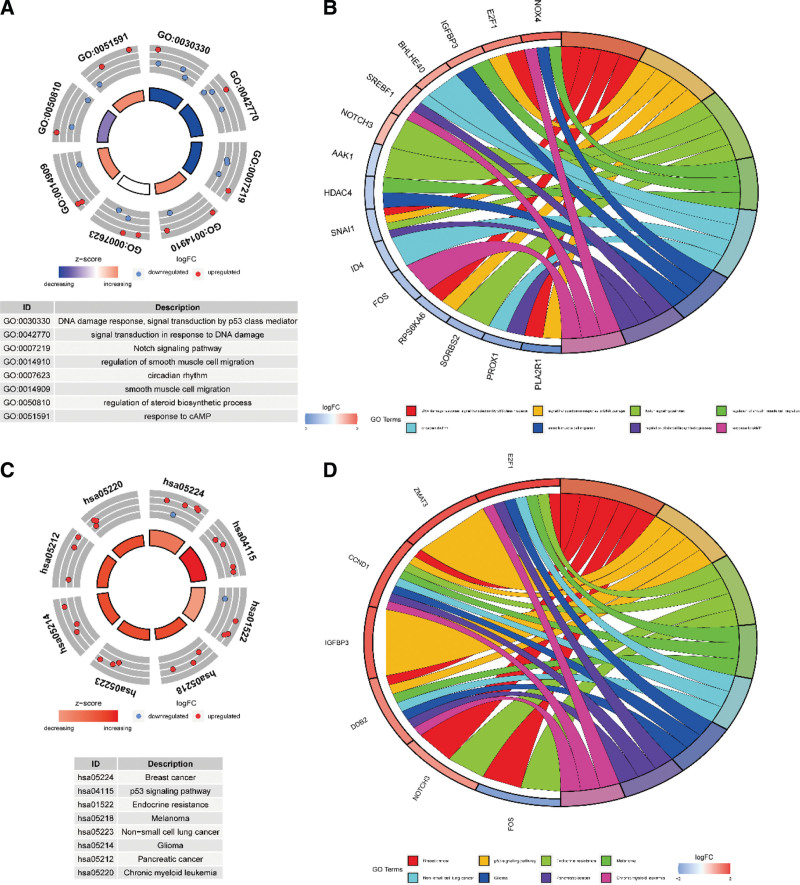
Gene functional enrichment analysis of SRGs. SRGs = senescence-related differentially expressed genes.

We further explore the related functions and pathways of SRGs through GO term and KEGG analyses. GO enrichment analysis revealed that SRGs were mainly involved in “DNA damage response, signal transduction by p53 class mediator,” “signal transduction in response to DNA damage” and “Notch signaling pathway” (Fig. 2A and B). KEGG enrichment analysis suggested that these genes were mainly enriched in “p53 signaling pathway” and “Endocrine resistance” (Fig. 2C and D).

### 3.3. Construction of a senescence-related prognostic model

**Figure 3.**
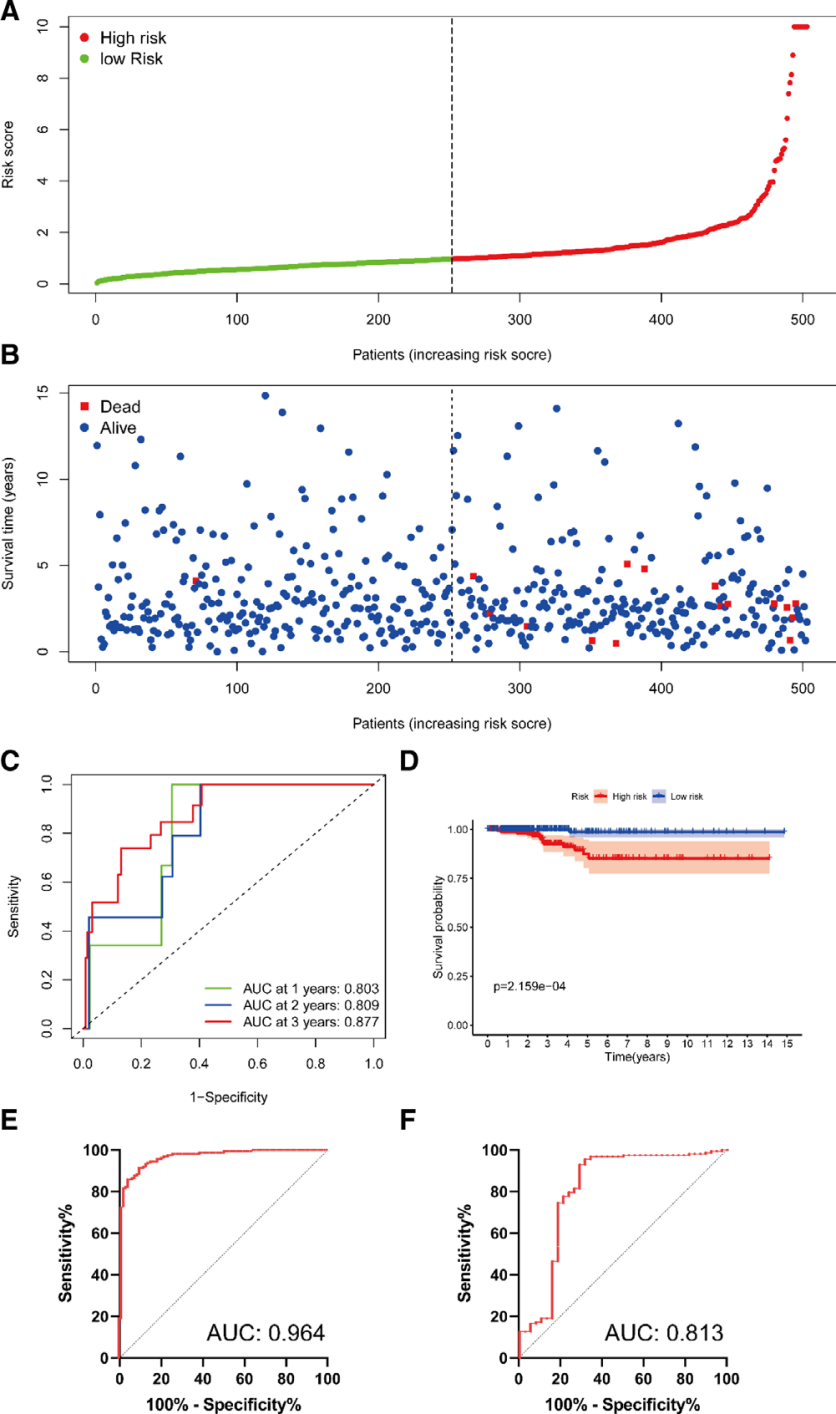
(A-B) The assessment of prognostic model in efficiency and clinical value, (C-D) The results of ROC curve (C) and K-M curve (D) in TCGA cohort, (E-F) The results of ROC curve in GPL570 (E) and GPL96 (F). K-M = Kaplan–Meier, ROC = receiver operating characteristic, TCGA = the cancer genome atlas.

Based on identification of SRGs, we searched for independent prognostic factors by univariate Cox regression analysis and found 9 genes, including histone deacetylase 4, E2F1, adaptor-associated kinase 1, SNAI1, mitogen-activated protein kinase 6, PLA2R1, sorbin, and SH3 Domain Containing 2, ubiquitin domain containing protein 1 and inhibitor of differentiation 4 (Table 1). We further analyzed these SRGs by multivariate Cox regression analysis and identified 3 genes, containing E2F1, SNAI1, and PLA2R1 (Table 2). Therefore, prognostic model was constructed to predict OS based on the risk score = e ^(−0.225^ * ^expression level of E2F1 + 0.060^ * ^expression level of SNAI1 + 0.095^ * ^expression level of PLA2R1)^. Then PTC patients were divided into a low-risk group (n = 252) and a high-risk group (n = 251) according to the median value (cutoff point = 0.969) of risk score in TCGA cohort (Fig. 3A and B).

**Table 1:** Univariate Cox regression analysis of senescence-related differentially expressed genes.

id	HR	HR.95L	HR.95H	*P* value
HDAC4	2.887388	1.579491	5.278291	.000571
E2F1	0.671618	0.483021	0.933855	.017939
AAK1	1.262871	1.034327	1.541913	.021945
SNAI1	1.094821	1.049359	1.142253	2.83E-05
MAP3K6	0.840609	0.718832	0.983016	.029668
PLA2R1	1.161602	1.072564	1.258032	.000232
SORBS2	1.041097	1.006804	1.076558	.018435
UBTD1	0.909538	0.830227	0.996426	.041662
ID4	1.006522	1.002194	1.010868	.003109

AAK1 = adaptor-associated kinase 1, E2F1 = E2F transcription factor 1, HDAC4 = histone deacetylase 4, HR = hazard ratio, ID4 = inhibitor of differentiation 4, MAP3K6 = mitogen-activated protein kinase 6, PLA2R1 = phospholipase A2 receptor 1, SNAI1 = snail family transcriptional repressor 1, SORBS2 = sorbin and SH3 Domain Containing 2, UBTD1 = ubiquitin domain containing protein 1.

**Table 2:** Multivariate Cox regression analysis of senescence-related differentially expressed genes.

id	coef	HR	HR.95L	HR.95H	*P* value
E2F1	−0.22452	0.798903	0.583946	1.092987	.160332
SNAI1	0.060488	1.062355	1.010208	1.117193	.018499
PLA2R1	0.09483	1.099472	0.993147	1.21718	.067633

E2F1 = E2F transcription factor 1, HR = hazard ratio, PLA2R1 = phospholipase A2 receptor 1, SNAI1 = snail family transcriptional repressor 1.

### 3.4. Diagnostic and prognostic value of prognostic model

ROC curves were constructed to assess diagnostic value of prognostic model. The AUC of TCGA cohort was 0.803, 0.809, and 0.877 at 1, 2, and 3 years, respectively (Fig. 3C), which showed good diagnostic efficiency. K-M curves were built to evaluate prognostic value of the model. *P* < .001 indicated significant statistical difference (Fig. 3D). The results of ROC curve from GPL570 and GPL96 further verified the reliability of this model (AUC = 0.964, 0.813 in GPL570 and GPL96, Fig. 3E and F).

### 3.5. Clinical significance of prognostic model

**Figure 4.**
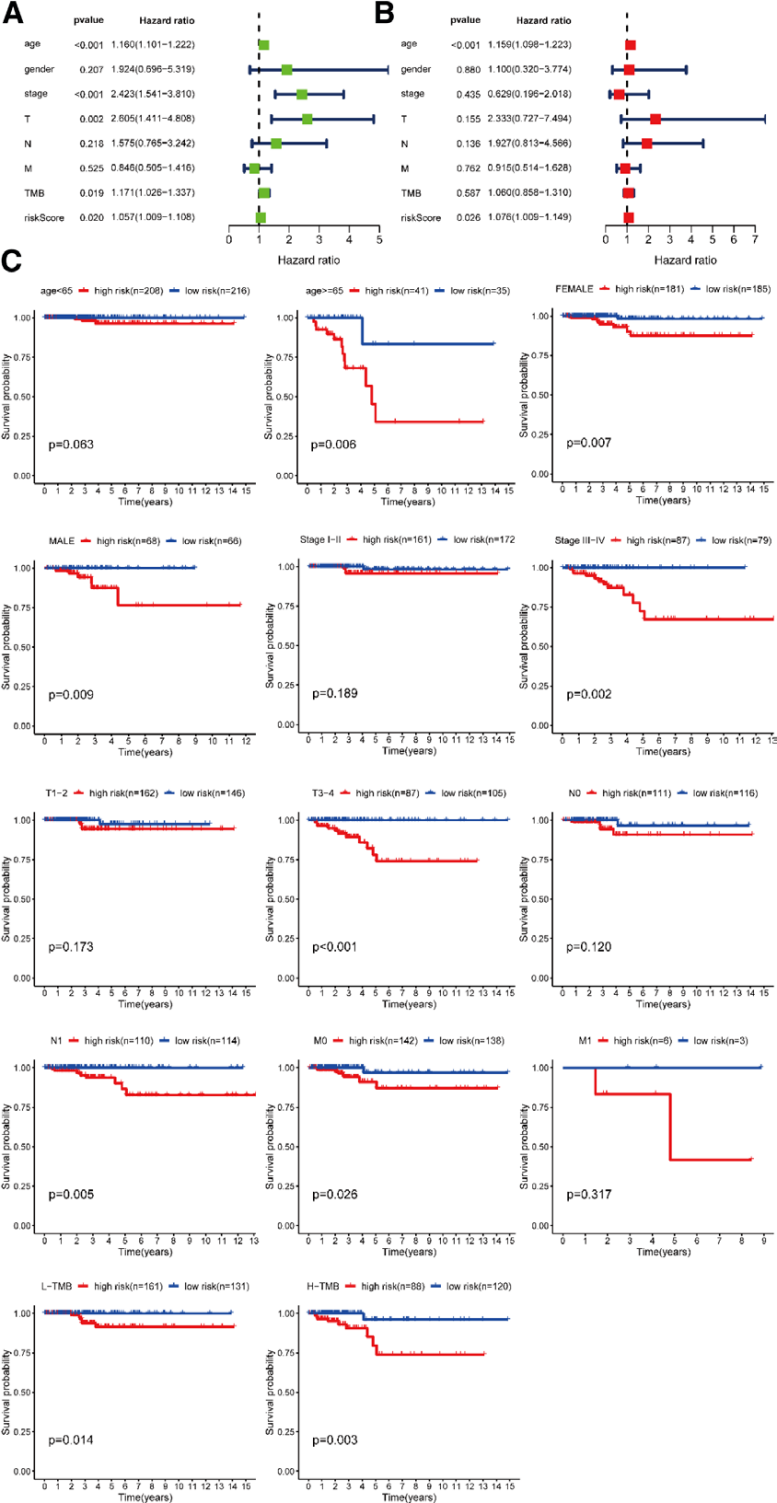
Independent and stratified prognostic analysis for clinical parameters.

On the 1 hand, we used univariate Cox regression analysis to observe the possibility of clinical parameters and riskScore as independent risk factors (see Table S4, Supplemental Digital Content, http://links.lww.com/MD/J82, which illustrates univariate and multivariate-Cox regression analysis of clinical parameters and risk score). We found that age, stage, T stage and risk score had the potential for prognosis (Fig. 4A). We further applied multivariate Cox regression analysis for these factors and also observe age and risk score were statistically different (Fig. 4B). On the 1 hand, stratified analysis of clinical parameters manifested that prognostic model performed well in most subgroups, including Female, Male, N1, M0, and L-tumor mutation burden (TMB), especially in grade and stage higher subgroup, such as age > = 65, Stage III − IV, T3 − 4, H-TMB (Fig. 4C).

### 3.6. Tumor immune infiltration analysis

**Figure 5.**
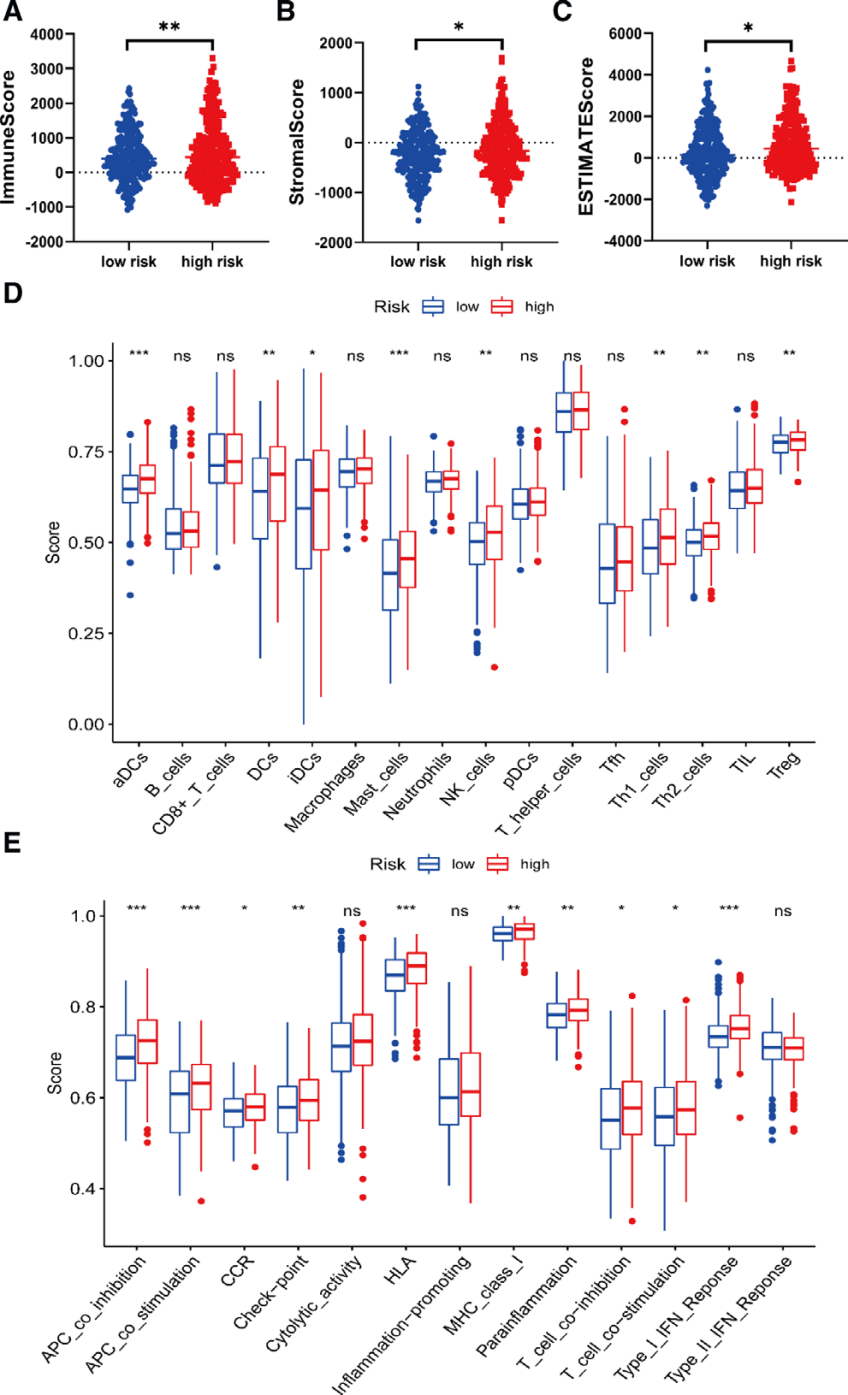
Immune infiltration analysis between low-risk and high-risk groups.

The Stromal score, Immune score and ESTIMATE score for TCGA cohort were compared between low-risk and high-risk groups. The results showed that high-risk group had a higher Stromal score (*P* < .05), Immune score (*P* < .01) and ESTIMATE score (*P* < .05) compared to low-risk group (Fig. 5A–C), which suggested that immune infiltration was closely related to the prognostic model. Meanwhile, single-sample gene set enrichment analysis further revealed that high-risk group had a more prominent immune infiltration in diverse immune cell subpopulations, associated functions or pathways, including DCs, Mast cells, NK cells, Th1 cells, Th2 cells, Treg cells, APC co-inhibition, APC co-inhibition, T cell co-inhibition, T cell co-stimulation, Type I IFN Reponse (Fig. 5D and E).

### 3.7. Expression levels of E2F1, SNAI1, and PLA2R1

**Figure 6.**
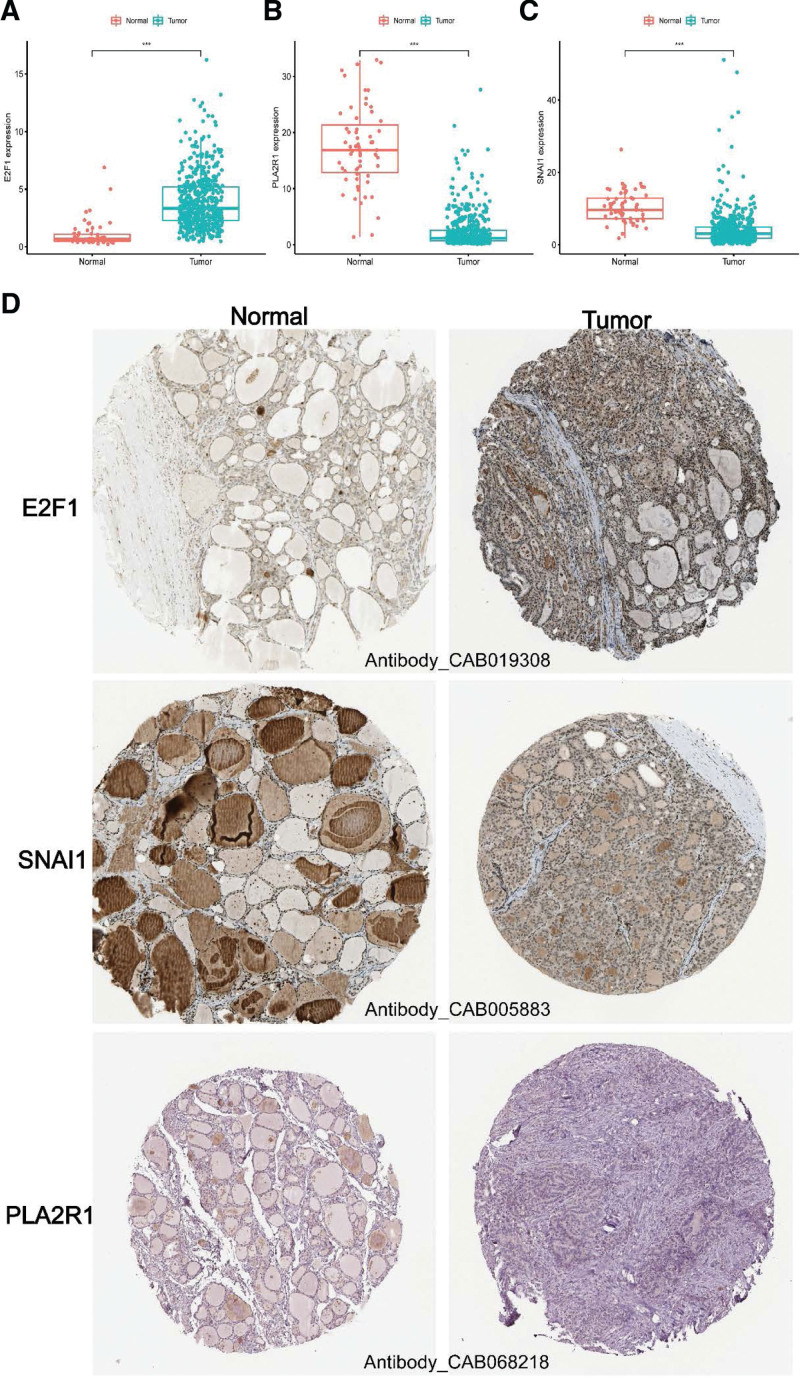
The expression levels of E2F1, SNAI1 and PLA2R1. E2F1 = E2F transcription factor 1, PLA2R1 = phospholipase A2 receptor 1, SNAI1 = snail family transcriptional repressor 1.

Finally, we analyzed the mRNA and protein expression levels of E2F1, SNAI1, and PLA2R1 for TCGA cohort between normal and PTC patients. We observed that the levels of SNAI1 and PLA2R1were higher, while the levels of E2F1 were lower in PTC group compared with normal group (Fig. 6A–D).

## 4. Discussion

As far as we know, we are the first to combine a large number of GEO platform datasets to expand the sample size and enhance the credibility of the results. Firstly, we obtained 3405 DEGs from TCGA cohort, 8045 DEGs from GPL570 and 5635 DEGs from GPLE96. Then we looked for SRGs co-expressed by the above DEGs and 297 senescence-related genes. We found 27 SRGs in total, most of them were enriched in “DNA damage response, signal transduction by p53 class Mediator,” “Signal transduction in Response to DNA damage,” “p53 signaling Pathway” and “Endocrine Resistance,” which further verified that SRGs played an important regulatory role in cellular senescence. Based on SRGs, we constructed a prognostic risk model consisting of E2F1, SNAI1, and PLA2R1. E2F1 regulated transcription of s-phase cyclins and genes required for DNA replication, DNA repair, and apoptosis. E2F1 was the prototype and most studied member of the E2F family, whose activity was tightly controlled by DNA damage checkpoints to regulate cell cycle progression and initiate programmed cell death when needed.^[21]^ Recent studies have shown that E2F1 mediated the proliferation and metastasis process of thyroid cancer,^[22,23]^ and simvastatin had a good pharmacological effect on the elimination of this process.^[24]^ SNAI1 was the first and most deeply studied e-cadherin transcriptional suppressor, and e-cadherin was a marker of epithelial-mesenchymal transformation encoded by epithelial gene Cadherin 1.^[25]^ SNAI1 directly bound to the E-box present in the Cadherin 1 promoter to transcriptionally inhibit its expression. On the other hand, SNAI1 also acted as a transcriptional activator to increase other epithelial-mesenchymal transformation transcription factors.^[26]^ It has been reported that SNAI1 expression suppressed cellular senescence in a variety of cancer cells and delayed the progression and metastasis of cancer.^[27]^ SNAI1 has also been confirmed to be involved in the migration and invasion of thyroid cancer.^[28]^ SNAI1 with multiple functions was worthy of further discussion and research. As in our data, PLA2R1 was downregulated in a number of other cancers and showed tumor suppressive activity.^[29]^ PLA2R1 was a 180 kDa transmembrane glycoprotein expressed in human foot cells, which was widely studied and reported in the membranous nephropathy,^[30]^ but the relevant research of PLA2R1 in thyroid cancer remains unclear.

Next, we continue to explore the clinical value of prognostic model. In general, the AUC followed the criteria: 0.50 to 0.60 = fail, 0.60 to 0.70 = poor, 0.70 to 0.80 = fair, 0.80 to 0.90 = good and 0.90 to 1 = excellent. The AUC of our model was 0.803, 0.809, and 0.877 at 1, 2, and 3 years respectively, which showed good predictive ability. And the effectiveness of the model has been verified by GPL570 and GPL96 (AUC = 0.964, 0.813, respectively). In addition, *P* = 2.159 E − 04 of K-M curve drawn by this model was far <0.01, which was statistically significant. Univariate and Multivariate Cox regression analysis were also used to further evaluate the potential of risk score as a risk factor. *P* < .05 was also observed, again proving the superiority of the model. Surprisingly, stratified analysis of clinical parameters found that the model had better significance for higher age, stage, grade and TMB groups, such as age > = 65, Stage III − IV, T3 − 4, H-TMB, while there was no statistical difference in age < 65, Stage I − II and T1 − 2 subgroups.

In addition, we analyzed the indexes related to immune infiltration in the low-risk group and the high-risk group, and unexpectedly found that the high-risk group showed significant enhancement of immune cell infiltration, which was a good attempt to evaluate immune infiltration by using the prognostic model based on SRGs.

## Author contributions

**Data curation:** Tingting Wen.

**Formal analysis:** Tingting Wen.

**Investigation:** Tingting Wen.

**Resources:** Shuang Guo.

**Supervision:** Shuang Guo.

**Validation:** Shuang Guo.

**Writing – original draft:** Tingting Wen.

**Writing – review & editing:** Shuang Guo.

## Supplementary Material








