# Supplementary material for: Bioinformatics analysis of the prognostic and clinical value of senescence-related gene signature in papillary thyroid cancer
Source: Medicine (Baltimore). 2023 Jun 2;102(22):e33934. doi: 10.1097/MD.0000000000033934 (PMC10238039; doi:10.1097/MD.0000000000033934)
Supplement: Supplementary file 1 [file medi-102-e33934-s001.pdf]

Table S1 Baseline data of papillary thyroid cancer patients from the The Cancer Genome Atla database.

clinical parameters	TCGA cohort
**No. of pateints**	507
**Age (years/median)**	47
**Sex (%)**	
**Female**	371
**Male**	136
**Stage(%)**	
Ⅰ	285
Ⅱ	52
Ⅲ	113
Ⅳ	55
**Unknown**	2
**T (%)**	
**T1**	144
**T2**	167
**T3**	171
**T4**	23
**Unknown**	2
**N (%)**	
**N0**	231
**N1**	226
**Unknown**	50
**M (%)**	
**M0**	283
**M1**	9
**Unknown**	215
**TMB (median)**	0.382
**OS （days/median）**	1211

TCGA= The Cancer Genome Atlas, TMB= tumor mutation burden, OS= overall survival
