# Supplementary material for: Bioinformatics analysis of the prognostic and clinical value of senescence-related gene signature in papillary thyroid cancer
Source: Medicine (Baltimore). 2023 Jun 2;102(22):e33934. doi: 10.1097/MD.0000000000033934 (PMC10238039; doi:10.1097/MD.0000000000033934)
Supplement: Supplementary file 3 [file medi-102-e33934-s003.pdf]

Table S3 27 overlap genes of GPL570、GPL96、The Cancer Genome Atlas and senescence-related genes.

PLA2R1
ZMAT3
RPS6KA6
BHLHE40
HDAC4
SNAI1
DDB2
ID4
PROX1
CCND1
ALOX15B
SREBF1
E2F1
IGFBP6
FOS
IRF5
LGALS3
SORBS2
IGFBP3
UBTD1
TNFSF15
EPHA3
NOX4
MAP3K6
SOX5
AAK1
NOTCH3
