# Supplementary material for: Bioinformatics analysis of the prognostic and clinical value of senescence-related gene signature in papillary thyroid cancer
Source: Medicine (Baltimore). 2023 Jun 2;102(22):e33934. doi: 10.1097/MD.0000000000033934 (PMC10238039; doi:10.1097/MD.0000000000033934)
Supplement: Supplementary file 4 [file medi-102-e33934-s004.pdf]

Table S4 Univariate- and multivariate- Cox regression analysis of clinical parameters and riskScore.

id	HR	HR.95L	HR.95H	pvalue	id	HR	HR.95L	HR.95H	pvalue
age	1.160268	1.101311	1.222383	2.31E-08	age	1.15883	1.097657	1.223413	9.97E-08
gender	1.923689	0.695769	5.318687	0.207351	gender	1.099677	0.320452	3.773699	0.87995
stage	2.422916	1.540754	3.810162	0.000127	stage	0.628797	0.195944	2.017847	0.435463
T	2.604554	1.410821	4.808335	0.002212	T	2.333401	0.726518	7.494324	0.15465
N	1.574846	0.765088	3.241642	0.217575	N	1.926569	0.81286	4.566182	0.136391
M	0.845963	0.505286	1.416334	0.524651	M	0.914801	0.514189	1.627533	0.761929
TMB	1.171421	1.026277	1.337092	0.019064	TMB	1.060306	0.858361	1.309762	0.586995
riskScore	1.057172	1.008915	1.107737	0.019686	riskScore	1.076448	1.008632	1.148825	0.026498

HR= hazard ratio, TMB= tumor mutation burden
